# *ZMYND10* - Mutation Analysis in Slavic Patients with Primary Ciliary Dyskinesia

**DOI:** 10.1371/journal.pone.0148067

**Published:** 2016-01-29

**Authors:** Małgorzata Kurkowiak, Ewa Ziętkiewicz, Agnieszka Greber, Katarzyna Voelkel, Alina Wojda, Andrzej Pogorzelski, Michał Witt

**Affiliations:** 1 Department of Molecular and Clinical Genetics, Institute of Human Genetics, Polish Academy of Sciences, Poznań, Poland; 2 International Institute of Molecular and Cell Biology, Warsaw, Poland; 3 Department of Pneumology and Cystic Fibrosis, Institute of Tuberculosis and Lung Diseases, Rabka Zdrój, Poland; National Eye Institute, UNITED STATES

## Abstract

Primary ciliary dyskinesia (PCD) is a rare recessive disease with a prevalence of 1/10,000; its symptoms are caused by a kinetic dysfunction of motile cilia in the respiratory epithelium, flagella in spermatozoids, and primary cilia in the embryonic node. PCD is genetically heterogeneous: genotyping the already known PCD-related genes explains the genetic basis in 60–65% of the cases, depending on the population. While identification of new genes involved in PCD pathogenesis remains crucial, the search for new, population-specific mutations causative for PCD is equally important. The Slavs remain far less characterized in this respect compared to West European populations, which significantly limits diagnostic capability. The main goal of this study was to characterize the profile of causative genetic defects in one of the PCD-causing genes, *ZMYND10*, in the cohort of PCD patients of Slavic origin. The study was carried out using biological material from 172 unrelated PCD individuals of Polish origin, with no causative mutation found in nine major PCD genes. While none of the previously described mutations was found using the HRM-based screening, a novel frameshift mutation (c.367delC) in *ZMYND10*, unique for Slavic PCD population, was found in homozygous state in two unrelated PCD patients. Immunofluorescence analysis confirmed the absence of outer and inner dynein arms from the ciliary axoneme, consistent with the already published *ZMYND10*-mutated phenotype; cDNA analysis revealed the lack of *ZMYND10* mRNA, indicating nonsense-mediated decay of the truncated transcript.

## Introduction

Primary ciliary dyskinesia (PCD, OMIM id: 242650), one of the major ciliopathies, is a rare (with a prevalence of 1/10,000), genetically heterogeneous autosomal recessive disease, caused by the abnormal structure and/or function of motile cilia [1–2]. To date, mutations in 29 genes have been found to underlie PCD pathogenesis in different families (for a recent review see Kurkowiak et al, 2015) [3]. Genotyping already known PCD-related genes (further referred to as PCD genes) explains the genetic basis in 60–65% of cases, depending on the population [4–5]. While identification of new genes involved in PCD pathogenesis remains crucial, a search for new, population-specific mutations causative for PCD is equally important. The Slavs remain far less characterized in this respect compared to West European populations, which significantly limits the routine diagnostic potential in this part of the continent.

A combined outer and inner dynein arms defect (ODA/IDA defect) is the most prevalent aberration of the ciliary ultrastructure observed in PCD [4]. This particular defect is also characteristic for PCD patients with mutations in the *ZMYND10* gene [6–7]. *ZMYND10* (zinc finger, MYND-domain-containing 10, NM_015896.2), located in chromosome 3p21, encodes the tumor suppressor protein, recently found to be involved in the ciliogenesis and responsible for the cytoplasmic preassembly of ciliary structure elements (ODA and IDA) [6]. Fourteen various *ZMYND10* mutations, distributed along the whole coding sequence, have been found so far in ~20 unrelated PCD patients from non-Slavic populations [6–7]. Here, we performed genetic screening for *ZMYND10* mutations in a group of Polish PCD patients preselected for the lack of mutations in nine major PCD genes.

## Materials and Methods

### Patients and Families with PCD

The study was performed in the group of 172 PCD preselected patients with no causative mutation found in nine major PCD genes (*DNAH5*, *DNAI1*, *RSPH4A*, *RSPH9*, *SPAG1*, *LRRC6*, *DNAI2*, *CCDC39*, *CCDC40*); these patients were a subgroup of the cohort of ~270 PCD families, whose genetic material is deposited in the authors’ laboratory. All patients fulfilled the clinical diagnostic criteria of PCD (neonatal respiratory distress, recurrent upper and lower respiratory tract infections, sinusitis, bronchiectasis, otitis media, in some left-right body asymmetry abnormalities-mainly *situs inversus*, low nNO level) [5,8]; data on the structural and/or functional defects of the respiratory cilia, identified by transmission electron microscopy (TEM) and/or high-speed video microscopy analyses, were available for the majority of patients. Written informed consent was obtained from the participating patients and/or their legal representatives. The study was approved by the Bioethical Committee at the Adam Mickiewicz University in Poznań (KB 435–13).

### Nasal NO (nNO) Analysis

Nasal NO was measured according to ERS recommendations [9], using ozone-chemiluminescence NO analyzer (Sievers Nitric Oxide Analyzer NOA 280i; GE Analytical Instruments, Boulder, USA; measurement range of 0.5-500ppm, detection limit 0.5 ppb, rise time 67 ms). Patients were not receiving antibiotics at the time of NO measurement.

### DNA Extraction

The genomic DNA was extracted from peripheral blood samples using a standard salting-out protocol. The DNA concentration and purity were evaluated by the measurement of 260/280 nm absorbance with the use of the Nanodrop (Thermo Scientific).

### Respiratory Epithelial Cell Samples

Respiratory epithelial cells were obtained by nasal brush biopsy performed by clinicians during routine check-ups. The cells were suspended in a cell culture medium, spread onto glass slides, air-dried and stored at –80°C until use; alternatively, the cells were stored in RNALater and used for RNA extraction.

### RNA Extraction and Reverse Transcription

Tissue from the patient’s nasal brush was treated with RNA Stabilization Reagent RNALAter^®^ (Life Technologies) and stored at 4°C. RNA was extracted from the nasal biopsy samples with the use of RNeasy^®^Plus Micro Kit (QIAGEN) for purification of total RNA from small samples according to the Qiagen protocol. A Wheaton glass homogenizer and recommended Buffer RLT Plus were used to homogenize the nasal brush sample; the RNA carrier (poly-A RNA, recommended by Qiagen) was added before homogenization to improve the recovery of total RNA. The homogenate was further processed using the column-centrifuge protocol. The amount and quality of RNA eluted were determined using 2100 Bioanalyser Agilent Technologies. Reverse transcription was performed according to the Enhanced Avian RT First Strand Synthesis Kit (SIGMA) protocol. The optimal reaction conditions were determined empirically (template RNA concentration 0.05 μg/ul, temperature 50°C and primers (random nanomeres).

### Primer Design and DNA Amplification

Selected *ZMYND* fragments were PCR-amplified from the genomic DNA or cDNA. PCR primers, designed with the use of Primer3 software, flanked whole exons or those exon fragments where the genotyped mutations were localized; to check if there were no SNPs in the primer binding sites, an SNPcheck v3 was used. The amplicon size for HRM analysis and sequencing, respectively, was 90–150 bp or 250–300 bp. A standard PCR amplification preceding dideoxy sequencing or cDNA analysis was carried out using: 0.4 ng/μl of DNA or cDNA template, 0.4 pM of each of the primers (forward and reverse), 0.12 mM deoxynucleoside triphosphates (Invitrogen) and a GoTaq polymerase kit (Promega); PCR reactions were conducted for 32 cycles with annealing temperatures of 60°C or 61°C. Real-time PCR reactions used in HRM analysis are described in one of the following sections. All primer pairs are listed in S1 Table.

### Dideoxy Sequencing

PCR products, purified with ExoSAP-IT reagent (Affymetrix), were sequenced using the dideoxy method (Laboratory of Molecular Biology Techniques, Adam Mickiewicz University, Poznań, Poland). The evaluation of sequencing results was performed using the Chromas software and Fasta Sequence Comparison tool (http://fasta.bioch.virginia.edu/fasta_www2/index.cgi). Any differences between analyzed and reference sequences were compared with the polymorphic variants data available in the Ensembl/dbSNP database. Any mutation identified was then described according to the nomenclature recommendations on the checklist for the description of sequence variants (http://www.hgvs.org/mutnomen/checklist.html). The numbering was that of the transcript 001 (Ensembl: ENSTT00000231749, RefSeq: NM_015896.2).

### Screening for Mutations

DNA samples were screened for the previously identified *ZMYND10* mutations using the high-resolution melt (HRM) technique. HRM allows detection of targeted sequence variations by recognition of different melting profiles of DNA fragments, and consists of two consecutive stages carried out in a single test-tube: the real-time PCR amplification of a DNA sample and the thermal dissociation step. The predicted curve characteristics of the amplicon sequences were assessed using UMelt and UMelt HETS software. Amplification was carried out in the 7900HT FastReal-Time PCR System (Applied Biosystems), using HOT FIREPol EvaGreen HRM Mix (Solis BioDyne) with 20 ng/μl DNA template and 0.4 pM primers (designed to amplify fragments in the 97–206 bp range). 40 cycles (denaturation temp. 95°C, annealing temp. 60°C and elongation temp. 72°C) were performed, immediately followed by melting profile analysis (HRM v2.0.2 software). Any samples exhibiting a change in the melting curve shape were sequenced using the dideoxy technique.

Single-strand conformational polymorphism (SSCP) analysis was performed to screen for unknown mutations. PCR-amplified segments (190–305 bp) were denatured and separated in 7% or 8% polyacrylamide (29:1) in 1xTBE; gels (optionally with ~2 M urea and 10% glycerol) were run for 20–40 h at 8-10W and at a room temperature or at 4°C.

### cDNA Analysis

To analyze the effect of the identified mutation (c.367delC) on mRNA, cDNA from the affected individual was amplified using primers located in the upstream *ZMYND10* exons (primers forward and reverse in exons 2 and 3, respectively; expected product length 152 bp). To assure the effectiveness of amplification, the reaction was performed in multiplex with primers located in exons 4 and 8 of the *GAPDH* (a housekeeping gene; expected product length 382 bp), exons 10 and 11 of the *CFTR* gene (expressed in bronchial epithelium; expected product length 301 bp), and exons 12 and 14 of the *LRRC6* (representing genes expressed in the ciliated epithelium; expected product length 224 bp). To prevent amplification from any contaminating genomic DNA, the amplicons were designed such that the length of the products amplified from the DNA template would exceed 1 kb (1.4 kb for the genomic DNA fragment delimited by primers located in *ZMYND10* exons 2 and 3). The annealing temperature was 61°C and the number of cycles was set at 31.

### Search for miRNA-binding Sites

The search for miRNA binding sites was performed using an online bioinformatics tool, miRDB (http://mirdb.org).

### Immunofluorescence Analysis

Respiratory epithelial cells on glass slides, stored at -80°C, were defrosted, washed with PBS and fixed by incubating with 4% PFA for 15 min. The samples were then treated with 0.2% Triton-X 100 for 10 min. to permeabilize the cell membranes, and incubated with 1% skim milk at 4°C overnight to block unspecific binding of the antibodies. Prepared samples were incubated with the primary antibodies for 2–3 h at room temperature, washed and incubated with the cross-absorbed secondary antibodies for 25 min at room temperature, and then with Hoechst 33342 (Sigma). The primary antibodies were IgGs directed against markers of various elements of the ciliary ultrastructure: acetylated α-tubulin (mouse monoclonal, SIGMA); α/β-tubulin (rabbit polyclonal, Cell Signaling Technology); DNAH5 (mouse monoclonal, generated [10]); DNALI1 (rabbit polyclonal, AVIVA); LRRC6 (rabbit polyclonal, AVIVA); CCDC39 (rabbit polyclonal, SIGMA). Immunofluorescence images were taken with Olympus Bx41 Microscope and processed with Isis software Metasystem International) and Adobe Creative Suite 4.

### Transmission Electron Microscopy (TEM)

Respiratory epithelial cells from bronchial biopsies were obtained during routine diagnostic procedures. The cells were fixed in 2.5% glutaraldehyde in 0.1 M sodium cacodylate buffer at 4°C, post-fixed in 1% osmium tetroxide, dehydrated and embedded in a mixture of propylene oxide and epoxy resin. Ultramicrotome sections were stained with Reynold’s lead citrate. Observations were performed at 16–30,000-fold magnification (Philips CM10).

## Results

### Screening for Mutations

The HRM technique was used to screen the selected Polish cohort of 172 PCD patients for the presence of seven PCD-causing *ZMYND10* mutations previously reported in the Caucasian population. None of the mutations was identified. The cohort was therefore subjected to SSCP-based analysis of the entire coding region of the *ZMYND10* gene (not shown). This analysis revealed the presence of seven sequence variants; the underlying sequence changes were identified by Sanger sequencing.

Four were known polymorphisms, also found in patients with mutations in another PCD gene (rs2301639, c.511-24C>T in intron 5, minor allele frequency in European population, MAF-Euro 12%; c.1105C>T; rs1989839, c.1306-30T>C in intron 11, MAF-Euro 13%; rs78166579, c.1323+39G>C in 3’UTR, MAF-Euro 4%; rs142613783, R369W in exon 10, MAF-Euro 1%).

Two other sequence changes were novel variants, each found in a single heterozygote (transversion in exon 8: c.744C>A; A248A; and transition in intron 9: c.999+4G>A). Both changes were conservatively assumed to represent non-deleterious variants, based on the following observations: i) no accompanying mutation on the second allele was identified; ii) the substitution in exon 8 did not change the amino acid; iii) the affected position (+4) in intron 9 was not a strictly conserved splice site.

**Fig 1:**
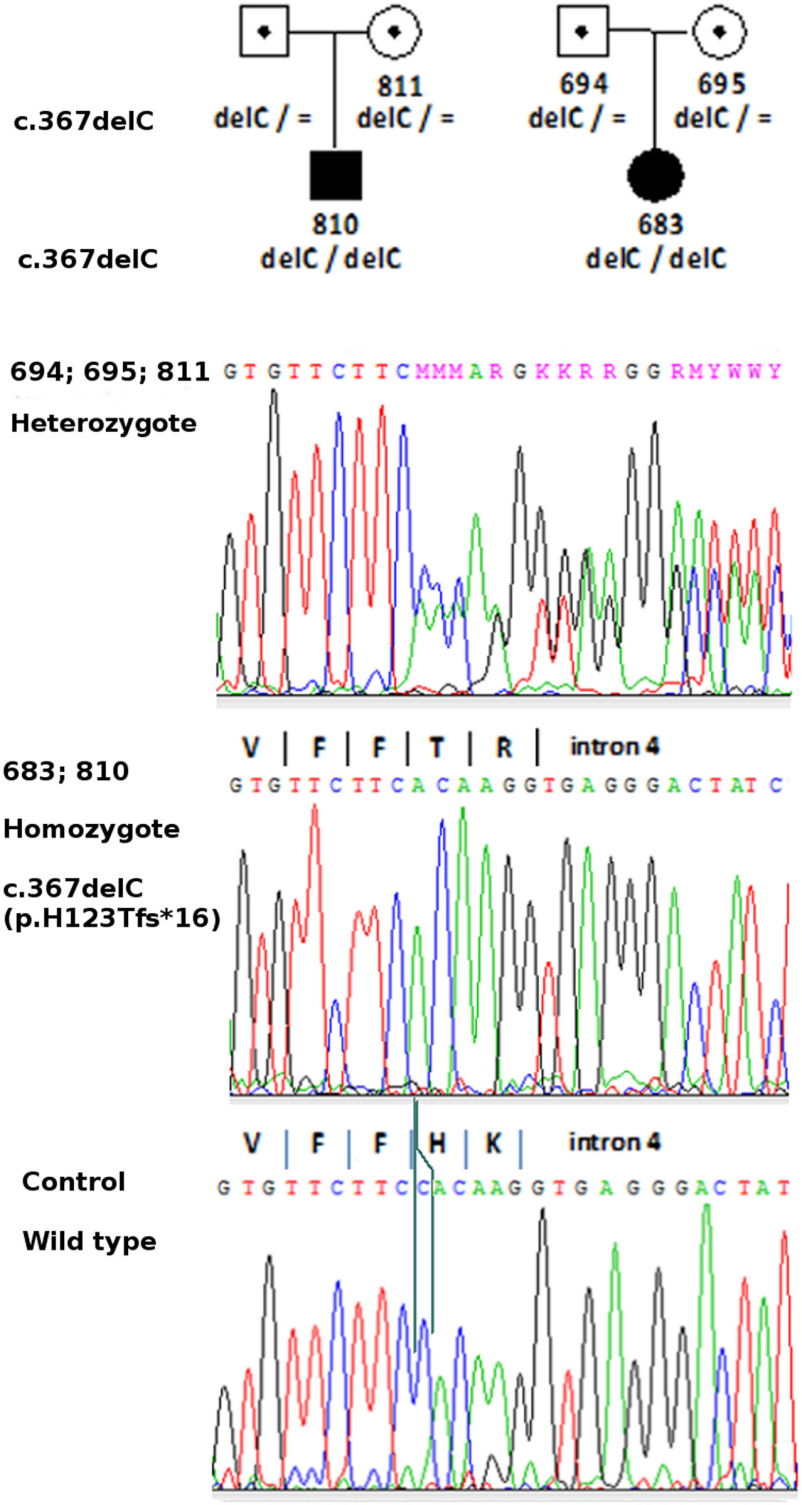
Analysis of the novel pathogenic allele in *ZMYND10* exon 4. Segregation of the mutated allele in two Polish PCD families confirmed recessive inheritance of the disease. Sequence analysis revealed the underlying frameshift mutation (c.367delC; p. H123Tfs*16). Patients and their parents’ numbers are indicated on pedigrees; filled symbols denote affected individuals, symbols with a point denote obligatory heterozygotes (carriers).

The only deleterious change was a frameshift mutation in exon 4 (c.367delC; p.H123Tfs*16), found in two unrelated homozygous patients (#683 and #810); incidentally, this mutation was also identified in #683 using the whole-exome sequencing (personal communication, EZ). The c.367delC was a novel mutation, not present in the polymorphic variants databases (dbSNP/Ensembl). Segregation analysis confirmed the recessive inheritance of the identified variant in both patients (Fig 1). To explain, why the c.367delC was not found by means of the HRM technique, the analysis was repeated using heterozygous samples (a 1:1 mixture of wild type and homozygous mutation samples, as well as obligatory carriers of the mutation—the parents of patient #683). A distinct HRM pattern was observed in all the heterozygotes (S1 Fig).

**Fig 2:**
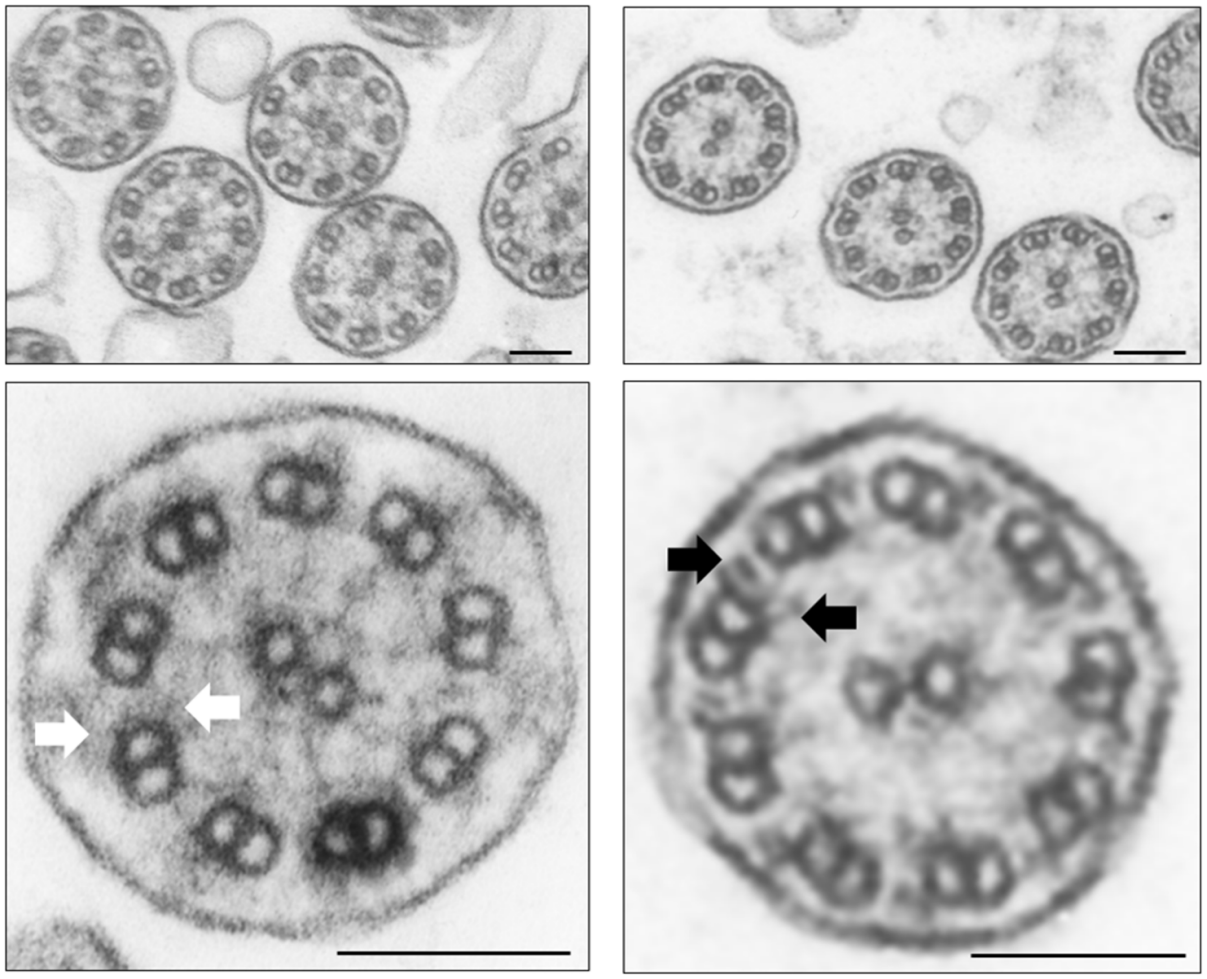
Transmission electron microscope analysis. Right panel, outer and inner dynein arms (ODA and IDA, black arrows) in the cross-section of cilia from the respiratory epithelium of a healthy individual. Left panel, lack of ODA and IDA (white arrows) in patient #683. Magnification 30,000; lower panel—enlarged view of a single cilium. Black scale bars represent 0,1 μm.

The clinical data for the *ZMYND10-*mutated subjects were consistent with the clinical diagnostic criteria for PCD (S2 Table). Both patients (#683 and #810) had very low nNO level (117 ppb and 23 ppb, respectively), compared to that in healthy subjects ranging from 450–760 ppb [11–14]. Patient #683 demonstrated *situs inversus totalis*, while patient #810 had *situs solitus*. TEM analysis of the ciliary cross-sections in respiratory epithelia samples obtained from both patients revealed the absence of both outer and inner dynein arms (ODA/IDA) (Fig 2).

Two families with the c.367delC mutations were from different regions of Poland and inquiries did not reveal any consanguinity within the families or across the families. To shed more light upon the origin of the mutation, the haplotype background composed of four neutral SNPs located within the gene downstream from the mutation (rs2301639, rs142613783, rs1989839, rs78166579) was analyzed. The c.367delC mutation was found to reside on the same haplotype variant (C-C-T-G) in both families affected (the distance between the mutation and the 3’-most markers was 1.2 kb). This haplotype was the most frequent one among four haplotype variants observed in the cohort analyzed (C-C-T-G: 84%, T-C-C-G: 10%, C-C-T-A: 3%, C-T-T-G: 3%; minor alleles are underlined). Based on the data available from our currently ongoing study (whole-exome sequencing of ~40 of the samples analyzed herein; data not shown), we found that the haplotype identity in c.367delC-carrying chromosomes (also shared by a quarter of the non-mutated chromosomes) extended ~217 kb and ~184 kb up- and downstream from the mutation, respectively (between rs1034405 and rs1138536).

### cDNA Analysis

**Fig 3:**
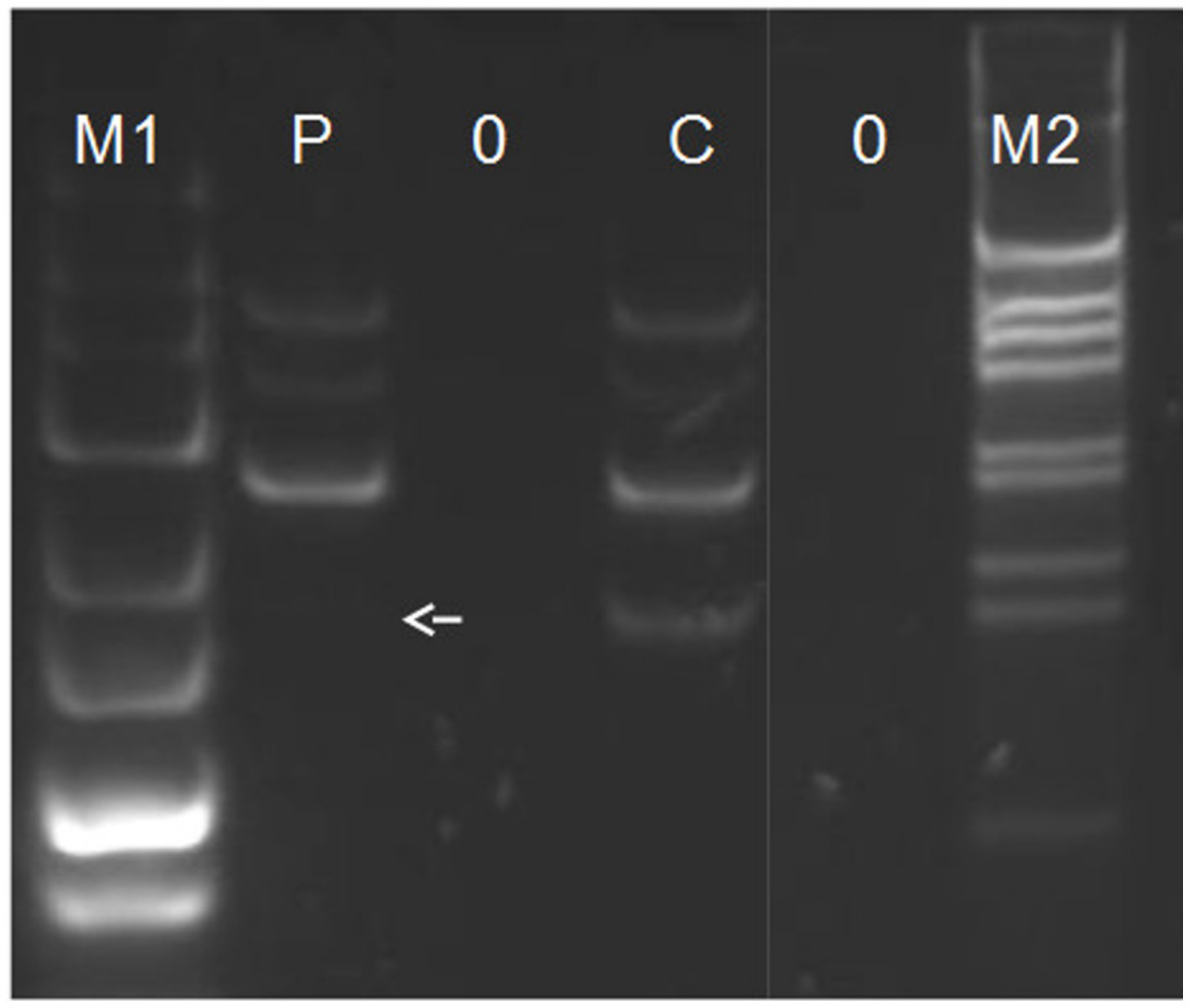
cDNA analysis. The multiplex PCR was carried out with the use of primer pairs located in the exons of *ZMYND10* (expected product length 152 bp), *LRRC6* (224 bp), *CFTR* (301 bp) and *GAPDH* (382 bp). PCR amplification products were separated on 4% NuSieve agarose gel (FMC BioProducts) in TBE buffer. M1: 25 bp ladder (Fermentas); M2: 1 kb ladder (LifeTechnologies), P: patient #683; C: healthy control; 0: blank control with water as a template. An arrow indicates the expected position of ZMYND10 product in patient #683.

The effect of the c.367delC mutation on the transcription efficiency of *ZMYND10* in respiratory epithelial cells was examined by cDNA analysis using *ZMYND10* primers designed to amplify a fragment of cDNA upstream from the identified mutation. To verify the proper performance of the PCR reaction, this reaction was performed in multiplex with *GAPDH* (housekeeping gene), *CFTR* (gene expressed in bronchial epithelium) and *LRRC6* (another gene expressed in ciliated cells). The amplification of *GAPDH*, *CFTR* and *LRRC6* was observed in both the healthy control and patient’s cDNA, while *ZMYND10* amplification was seen only in the control (Fig 3).

**Fig 4:**
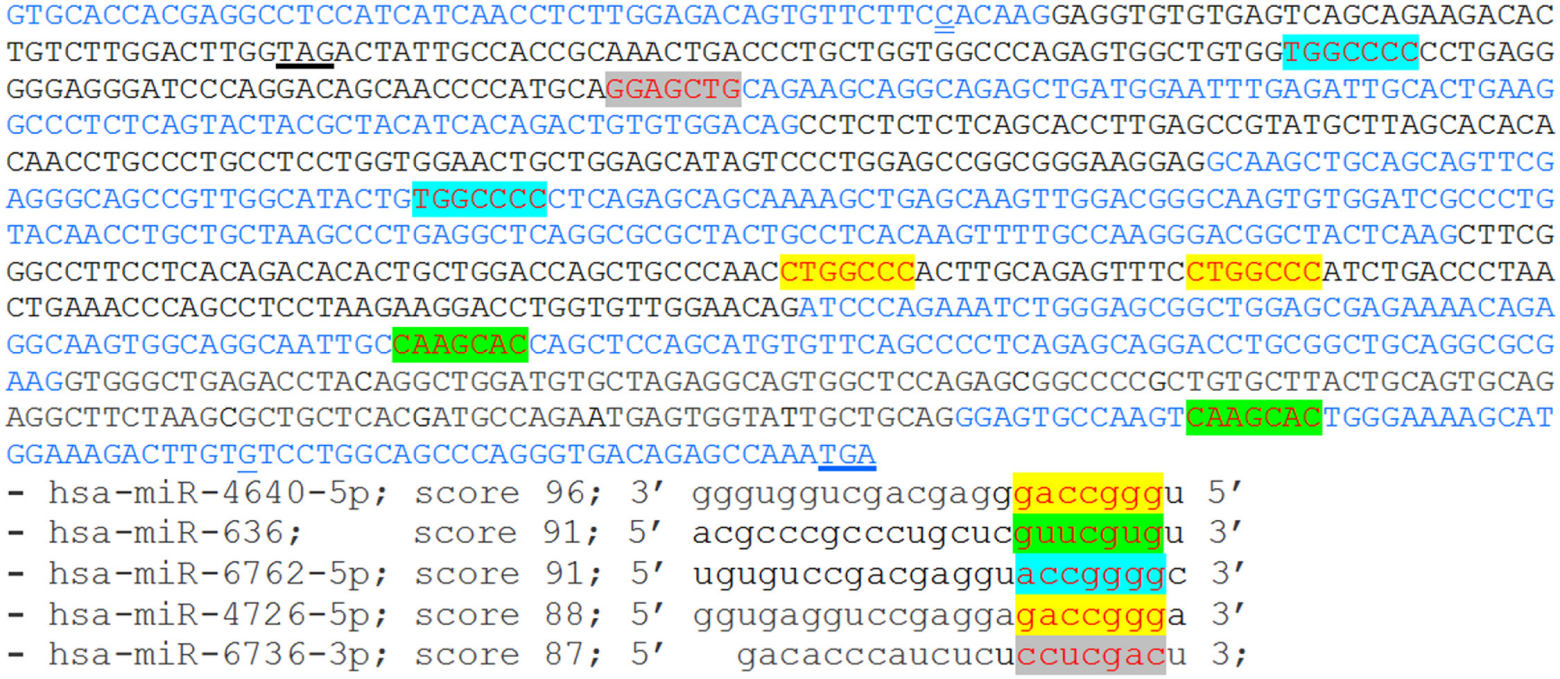
Potential miRNA target sites in *ZMYND10* mRNA. Potential miRNA target sites predicted using miRDB (database tab: ‘*Custom Prediction; Search for unconventional target sites in the coding region or 5'-UTR*’) are shown in the mRNA sequence downstream from the c.367delC mutation (spliced exons 4–12 in transcript 001 are shown in alternating blue and black colors; the site of the mutation is double underlined; position of the STOP codons—normal and premature—are underlined in bold). Seed sequences complementary to the highest scoring miRNAs (sequences shown below) are highlighted.

To explore the possibility that the level of mutated *ZMYND10* allele expression was regulated by miRNA-mediated surveillance mechanism [15], we searched the sequence downstream of the c.367delC mutation for the presence of miRNA target sites, using the bioinformatic tool miRDB available online, based on support vector machines (SVMs) and high-throughput training datasets (http://mirdb.org/miRDB) [16–17]. A computational target prediction algorithm assigned high scores to a number of target motifs for several miRNAs in the *ZMYND10* sequence downstream of the frameshifting deletion. The five best hits were scored between 96 and 87 (according to the SVMs description, the scores >80 are considered most likely to be real) (Fig 4), indicating that miRNA regulation of the *ZMYND10* gene is possible; however, the role of this mechanism in the elimination of the mRNA with mutation(s) that introduce premature termination codon remains to be investigated.

### Immunofluorescence Staining

**Fig 5:**
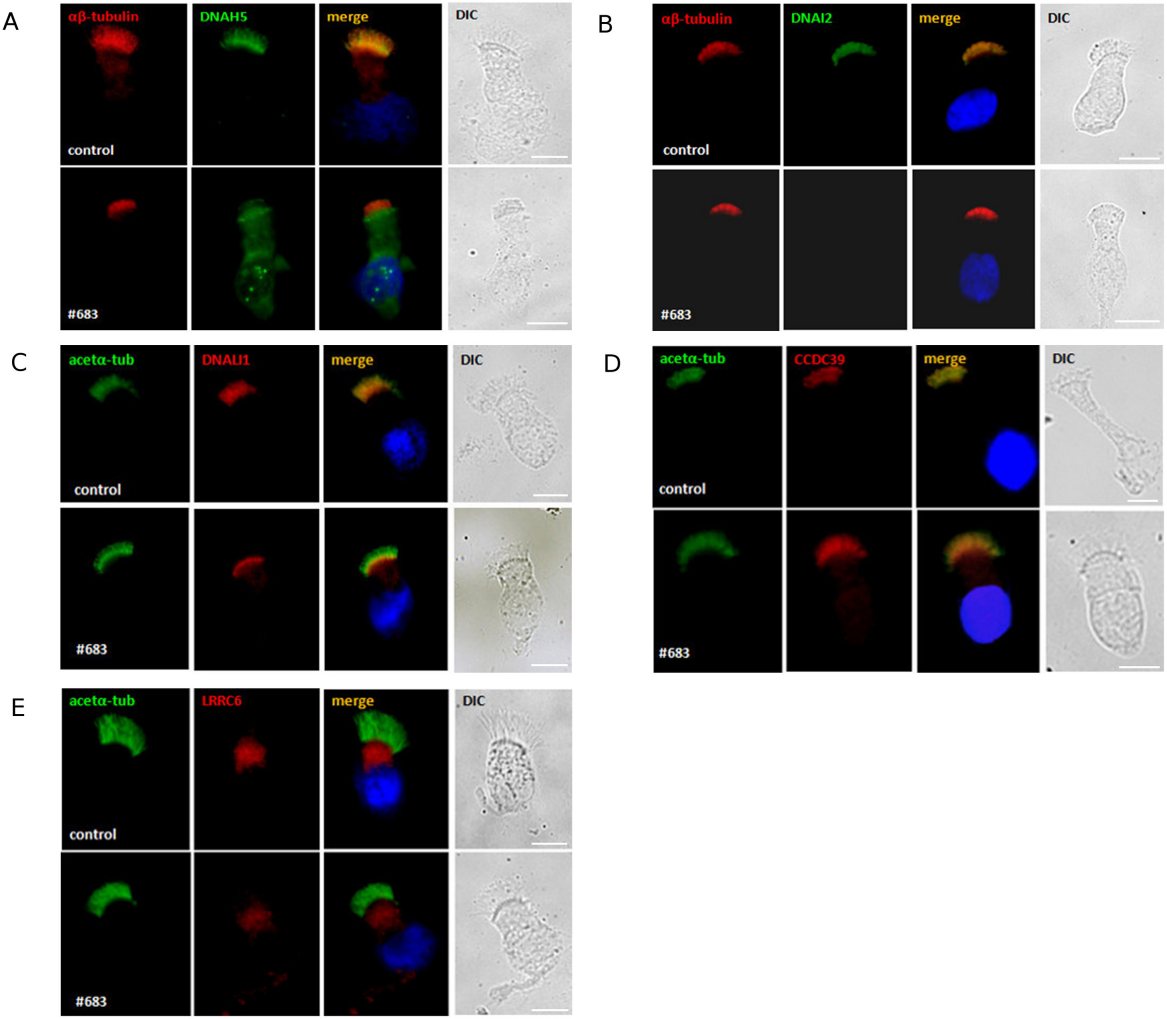
High-resolution immunofluorescence analysis of the subcellular localization of the ciliary ultrastructure markers. αβ-tubulin (red; in A) or acetylated α-tubulin (green; in B, C) were markers of microtubules; DNAH5 (green, in A) and DNAI2 (green, in B)–of outer dynein arms; DNALI1 (red, in C)–of inner dynein arms; CCDC39 (red, in D)–of nexin-dynein regulatory complex, and LRRC6 (red, in E)–of cytoplasmic assembly complex. DIC, differential interference contrast; nuclei were stained with Hoechst 33342 (blue). Respiratory epithelium cells from the control and from patient #683 are shown in upper and lower panels, respectively. White scale bars represent 10 μm.

The impact of the c.367delC mutation on the ciliary structure was assessed using immunofluorescence (IF) analysis. IF staining was performed in the respiratory epithelium samples from patient #683, using primary antibodies for markers of ODA (DNAH5, DNAI2), and IDA (DNALI1), microtubules (acetylated α-tubulin or αβ-tubulin), nexin-dynein regulatory complex (CCDC39) and cytoplasmic assembly complex (LCCR6) (Fig 5). The staining revealed the absence of DNAH5, DNAI2 and DNALI1 proteins from the cilia in the patient; DNAI2 was absent from the cell, DNAH5 was present in the cytoplasm and in the form of foci surrounding the nucleus, while DNALI1 was concentrated in the vicinity of basal bodies. Localization of and CCDC39 (in cilia), and of LRRC6 (in the apical cytoplasm) did not differ between the patient and the control.

## Discussion

**Fig 6:**
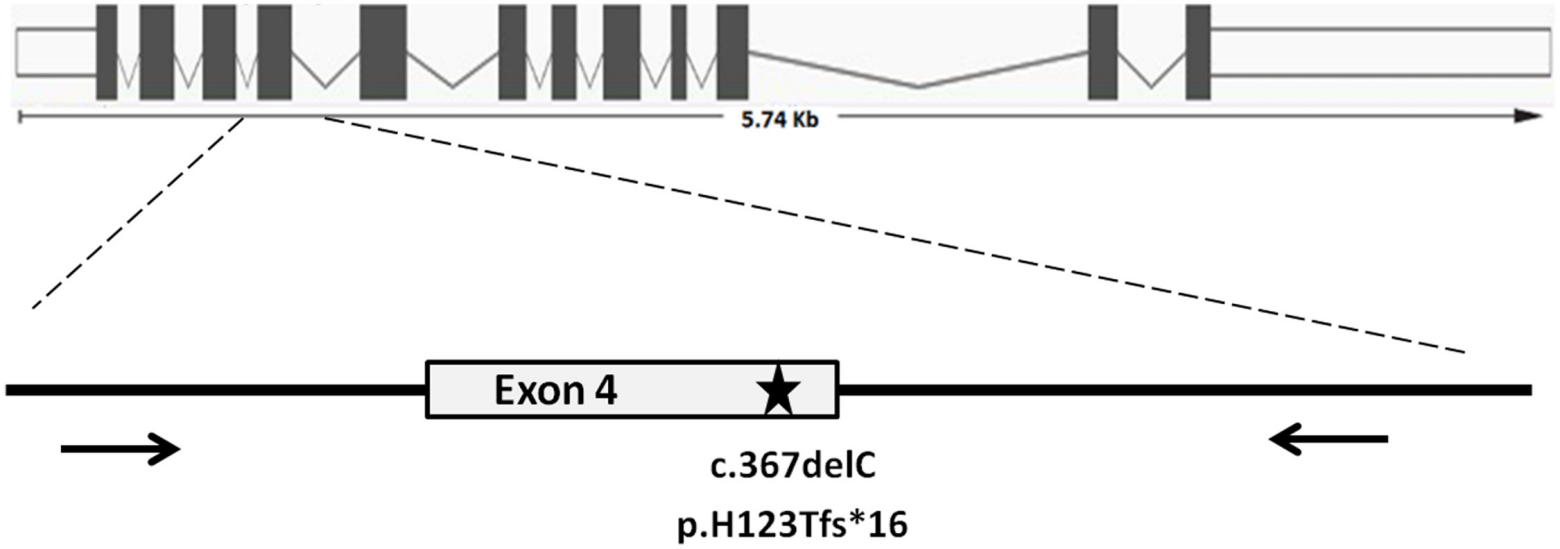
Localization of the c.367delC mutation and primers used to analyze *ZMYND10* exon 4. Upper panel—exon structure of the *ZMYND10* gene; lower panel—exon 4; an asterisk indicates position of the homozygous mutation found in patients #683 and #810; the short arrows depict approximate localization of the primers (forward and reverse) used for HRM and SSCP analysis

To date, 14 various *ZMYND10* mutations in PCD patients have been described in the literature [6–7]. Screening of the preselected group of Polish patients did not reveal any of these mutations, including c.47T>G, the most common variant of this gene, previously found in six families of European origin. Instead, a novel c.367delC frameshift mutation in exon 4 (Fig 6) was identified in two homozygous, unrelated Polish PCD patients from different regions of Poland. So far, this mutation appears to be restricted only to PCD patients of Slavic origin. The c.367delC is therefore added to the list of *ZMYND10* mutations (Table 1), confirming the lack of mutation predilection for any specific exons of the *ZMYND10* gene. Finding a single mutation in two families in the total cohort of over 270 Polish PCD families (i.e. including 172 patients screened for mutations in this study plus the remaining 98, in whom mutations in other genes had been previously identified) indicates that mutations in *ZMYND10*, accounting for ~0.7% of Polish PCD chromosomes, are not among the most frequent causes of PCD in Polish patients.

**Table 1:** *ZMYND10* mutations identified in different studies.

Mutation	Exon	Ethnic origin of patients	Number of families	References
**c.47T>G, p.V16Q**	1	Caucasians; North Europeans	1; 1^a^ 2^b^^,^^c^; 2^d^	[6,7]
c.65delT, p.F22Sfs*21	1	UK-Pakistani	1^d^	[7]
**c.85T>C, p.S29P**	1	Hispanic; French	1^d^; 1	[6]
**c.116T>C, p.L39P**	2	North European	1^b^	[7]
c.300delC, p.F101Sfs*38	3	Caucasian	1; 1^a^	[6]
c.367delC, p.H123Tfs*16	4	Polish	2	herein
c.486dupA, p.S163Ifs*20	5	Turkish	1^d^	[6]
c.589_590delTG, p.V198Gfs*13	6	North European	1^c^	[7]
c.608_609dupTC, p.T205Afs*3	7	Israeli	1^d^	[6]
c.683G>A; p.W228*	7	Turkish	1	[6]
**Exons 7–12 deletion**	7–12	Turkish	1^d^	[6]
**c.797T>C; p.L266P**	8	North European	1	[7]
c.967C>T, p.Q323*	9	Hispanic; French	1;1	[6]
c.1038_1039delAG, p.G347Qfs*30	10	Portugese	1	[6]
c.1096C>T, p.Q366*	10	Caucasian	1	[6]
**c.1136A>G, p.Y379C**	11	Israeli	1^d^	[6]

^a,b,c^Families with compound mutations; ^d^known consanguinity.

The total number of families examined was: >300 PCD [6] and 38 PCD preselected for ODA/IDA defect [7].

It is important to keep in mind that this could be an underestimate, as SSCP-based screening for sequence changes is never 100% efficient. However, using various electrophoretic conditions greatly increases the detection rate, and our long-standing experience with this technique allows us to assume that 80–85% of mutations are readily detected. Direct sequencing of the *ZMYND10* coding sequence (not shown), performed in ~60 randomly selected patients with no mutations identified using two screening techniques (HRM and SSCP), did not reveal any new variants. Interestingly, in the case of the homozygous c.367delC mutation, SSCP turned out to be more sensitive than HRM, whereby the deletion could be revealed only in the heterozygous state. While routine protocols for HRM analysis do not call for mixing samples with controls prior to the analysis, the case described here indicates that this may be a useful strategy for increasing the efficiency of HRM-based sequence variant detection.

Considering that *ZMYND10* mutations were found in 16 out of 570 families examined worldwide (300 in Zariwala et al, 2013 [6] and 270 herein), the overall contribution of this gene to the profile of genetic causes of PCD can be estimated at ~3%.

In both Polish families, c.367delC resided on the same background haplotype (C-C-T-G) composed of four frequent SNPs located within the *ZMYND10* gene. However, contrary to other populations, where different homozygous mutations had been found [6–7], no consanguinity between family members and across the two Polish families was reported in spite of the repeated enquiries that had been carried out following identification of the shared mutation. In addition, the C-C-T-G haplotype was the most frequent in the general Polish population (84%), rendering conclusions about the common versus independent origin of the mutation in both families difficult. Based on the data available from our currently ongoing study (whole-exome sequencing of ~40 of the samples analyzed herein), we found that the haplotype identity in c.367delC-carrying chromosomes extended at least ~217 kb and 184 kb up- and downstream from the mutation, respectively (between rs1034405 and rs1138536); the same haplotype was present in a quarter of the non-mutated chromosomes. While the independent origin of the c.367delC mutation in the ancestors of two analyzed Polish families could not be formally excluded, the shared background haplotype extending over 0.4 Mb favors the possibility that the mutation-carrying chromosomes are distantly related. Drawing further conclusions about the origin and age of the c.367delC mutation would require extensive genotyping of other markers, e.g. microsatellites, which was not the aim of this study.

Analysis of cDNA derived from the patient’s epithelial tissue from bronchial biopsy revealed the complete absence of *ZMYND10* mRNA. This indicated that a frameshift deletion resulting in the STOP codon in exon 5 (p. H123Tfs*16) could have triggered nonsense-mediated mRNA decay (NMD). As a result, instead of the expected truncated protein production, no polypeptide would be translated in the individual with the mutation.

Another mechanism for the elimination of mRNA containing premature termination codon has been proposed, whereby the coding region downstream of the mutation may be converted into the 3’UTR, which could contain binding sites for microRNAs [15]. This miRNA-mediated surveillance, involving accelerated mRNA deadenylation and translational inhibition, could eliminate mutated mRNAs that escape NMD or both NMD and miRNA-mediated repression could work together [15]. However, the significance of this pathway requires further research, as the microRNA control tends to be weak [18]. The presence of target motifs for several miRNA found in the *ZMYND10* sequence downstream of the frameshifting deletion suggests that the possibility of this mechanism being involved in the elimination of mutated *ZMYND10* mRNA should not be excluded.

The results of the TEM analysis of ciliated cells from the c.367delC homozygote were congruent with the previously described ZMYND10 mutation phenotype, i.e. ODA/IDA defect [6–7]. Consistently, DNAH5 and DNALI1 signals in the IF analysis were absent from the axonemes and retained in the cytoplasm, DNAI2 were completely absent from the cells.

Mislocalization of ODA components (DNAI2 and DNAI1) and IDA components (DNALI1 and DNAH7) have been previously shown in PCD individuals with mutations in *LRRC6*, encoding another cytoplasmic protein involved in the assembly of cilia [19–20]. Recently published studies have revealed interaction between the C-termini of ZMYND10 and LRRC6 [6–7]. Since mutation in either *ZMYND10* or *LRRC6* are sufficient to disturb ODA and IDA formation, the role of ZMYND10 and LRRC6 in the cytoplasmic assembly of dynein arms has been suggested [6–7]. It has been hypothesized that ZMYND10 and LRRC6 might be a part of a larger cytoplasmic protein complex (including other proteins potentially involved in PCD pathogenesis, LRRC50, KTU, DNAAF3, DYX1C1), necessary for the proper assembly of functional cilia. In our experiments, the cytoplasmic localization of LRRC6 protein was not affected in the *ZMYND10*-mutated cells. This suggests that while the interaction between LRRC6 and ZMYND10 is necessary for the proper assembly of dynein arms, the lack of one of these proteins may not be sufficient to disturb the formation of the assembly complex.

Finally, the axonemal localization of CCDC39, a part of the nexin-dynein regulatory complex (N-DRC), did not differ from that in the control, indicating that the deficient process of dynein arms assembly does not affect formation of the N-DRC complex. These results reaffirm the observation that the assembly of ciliary components requires participation of a complex, multi-protein machinery. This in turn warrants further studies to fully elucidate the role of all the protein factors involved in the assembly of cilia and to estimate their importance in the molecular basis of PCD.

## Conclusions

A novel c.367delC frameshift mutation was identified in two homozygous, unrelated PCD patients from different regions of Poland. So far, this mutation appears to be restricted only to PCD patients of Polish origin, confirming our earlier observation that the profile of PCD-causing mutations in Slavs differs from that reported in other European populations. Combined with the data from other populations, the overall contribution of *ZMYND10* mutations in PCD can be estimated as ~3%. The IF results of the ciliated cells from the patients affected reaffirm the earlier observation that *ZMYND10* mutations affect ODA and IDA assembly; further studies are required to fully elucidate the role of all the players involved in the assembly of cilia and to estimate their importance in the molecular basis of PCD.

## Supporting Information

S1 Fig Plots illustrating high resolution melting (HRM) profile of amplicons containing wild type allele and c.367delC variant. Green, red and purple (two runs each) denote, respectively: the wild type homozygous control, the c.367delC homozygous patient #683, an artificial heterozygote (1:1 mixed amplicons from the homozygous patient #810, and the control DNA). The melting patterns of two homozygotes were almost identical; therefore only heterozygotes were detectable in the HRM analysis.
(TIFF)

S1 Table List of primers. Upper- and lowercase indicate sequences in exons and introns, respectively; asterisks denote primers pairs used in multiplex PCR with the cDNA templates
(DOC)

S2 Table Clinical data for *ZMYND10* mutated patients. ppb, parts per billion.
(DOCX)

## References

[1] Kuehni CE, Frischer T, Strippoli MP, Maurer E, Bush A, Nielsen KG, et al Factors influencing age at diagnosis of primary ciliary dyskinesia in European children. *Eur Respir J* 2010; 36:1248–58. 10.1183/09031936.00001010 20530032

[2] Barbato A, Frischer T, Kuehni C, Snijders D, Azevedo I, Baktai G, et al Primary ciliary dyskinesia: a consensus statement on diagnostic and treatment approaches in children. *Eur Respir J* 2009; 34:1264–76. 10.1183/09031936.00176608 19948909

[3] Kurkowiak M, Zietkiewicz E, Witt M. Recent advances in primary ciliary dyskinesia genetics. *J Med Genet* 2015;52:1–9. 10.1136/jmedgenet-2014-102755 25351953 PMC4285891

[4] Zariwala M, Knowles M, Omran H. Genetic defects in ciliary structure and function. *Annu Rev Physiol* 2007; 69:423–50. 17059358 10.1146/annurev.physiol.69.040705.141301

[5] Stillwell P, Wartchow E, Sagel S. Primary Ciliary Dyskinesia in Children: A Review for Pediatricians, Allergists, and Pediatric Pulmonologists. *Pediatr Allergy*, *Immunol Pulmonol* 2011; 24:191–196. 22276227 10.1089/ped.2011.0099 PMC3255511

[6] Zariwala M, Gee H, Kurkowiak M, Al-Mutairi D, Leigh M, Hurd T, et al ZMYND10 is mutated in primary ciliary dyskinesia and interacts with LRRC6. *Am J Hum Genet* 2013; 93:336–45. 10.1016/j.ajhg.2013.06.007 23891469 PMC3738827

[7] Moore D, Onoufriadis A, Shoemark A, Simpson M, zur Lage P, de Castro S, et al Mutations in ZMYND10, a gene essential for proper axonemal assembly of inner and outer dynein arms in humans and flies, cause primary ciliary dyskinesia. *Am J Hum Genet* 2013; 93:346–56. 10.1016/j.ajhg.2013.07.009 23891471 PMC3738835

[8] Boon M, Smits A, Cuppens H, Jaspers M, Proesmans M, Dupont L, et al Primary ciliary dyskinesia: critical evaluation of clinical symptoms and diagnosis in patients with normal and abnormal ultrastructure. *Orphanet J Rare Dis* 2014; 9:1–10. 24450482 10.1186/1750-1172-9-11 PMC4016480

[9] Kharitonov S, Alving K, Barnes PJ. Exhaled and nasal nitric oxide measurements: recommendations. The European Respiratory Society Task Force. *Eur Respir J* 1997; 10:1683–93. 9230267 10.1183/09031936.97.10071683

[10] Omran H, Kobayashi D, Olbrich H, Tsukahara T, Loges NT, Hagiwara H, et al Ktu/PF13 is required for cytoplasmic pre-assembly of axonemal dyneins. *Nature* 2008; 456:611–6. 10.1038/nature07471 19052621 PMC3279746

[11] Karadag B, James AJ, Gültekin E, Wilson NM, Bush A. Nasal and lower airway level of nitric oxide in children with primary ciliary dyskinesia. *Eur Respir J*. 1999; 13:1402–5. 10445619 10.1183/09031936.99.13614069

[12] Wodehouse T, Kharitonov SA, Mackay IS, Barnes PJ, Wilson R, Cole PJ. Nasal nitric oxide measurements for the screening of primary ciliary dyskinesia. *Eur Respir J*. 2003; 21:43–7. 12570107 10.1183/09031936.03.00305503

[13] Struben VM, Wieringa MH, Mantingh CJ, Bommeljé C, Don M, Feenstra L, et al Nasal NO: normal values in children age 6 through to 17 years. *Eur Respir J*. 2005; 26:453–7. 16135727 10.1183/09031936.05.00015205

[14] Shoemark A, Wilson R. Bronchial and peripheral airway nitric oxide in primary ciliary dyskinesia and bronchiectasis. *Respir Med* 2009; 103, 700–706. 10.1016/j.rmed.2008.12.004 19117740

[15] Zhao Y, Lin J, Xu B, Hu S, Zhang X, Wu L. MicroRNA-mediated repression of nonsense mRNAs. *Elife* 2014; 3:e03032 10.7554/eLife.03032 25107276 PMC4359369

[16] Wang X, El Naqa IM. Prediction of both conserved and nonconserved microRNA targets in animals. *Bioinformatics* 2008; 24:325–332. 18048393 10.1093/bioinformatics/btm595

[17] Wang X. miRDB: a microRNA target prediction and functional annotation database with a wiki interface. *RNA* 2008; 14:1012–1017. 10.1261/rna.965408 18426918 PMC2390791

[18] Leung AK, Sharp PA. MicroRNA functions in stress responses. *Mol Cell* 2010; 40:205–15. 10.1016/j.molcel.2010.09.027 20965416 PMC2996264

[19] Kott E, Duquesnoy P, Copin B, Legendre M, Dastot-Le Moal F, Montantin G, et al Loss-of-function mutations in LRRC6, a gene essential for proper axonemal assembly of inner and outer dynein arms, cause primary ciliary dyskinesia. *Am J Hum Genet* 2012; 91:958–64. 10.1016/j.ajhg.2012.10.003 23122589 PMC3487148

[20] Horani A, Ferkol T, Shoseyov D, Wasserman M, Oren Y, Kerem B, et al LRRC6 mutation causes primary ciliary dyskinesia with dynein arm defects. *PLoS One* 2013; 8:e59436 10.1371/journal.pone.0059436 23527195 PMC3602302

