# Supplementary figures and images for: ZMYND10 - Mutation Analysis in Slavic Patients with Primary Ciliary Dyskinesia
Source: PLoS One. 2016 Jan 29;11(1):e0148067. doi: 10.1371/journal.pone.0148067 (PMC4732763; doi:10.1371/journal.pone.0148067)

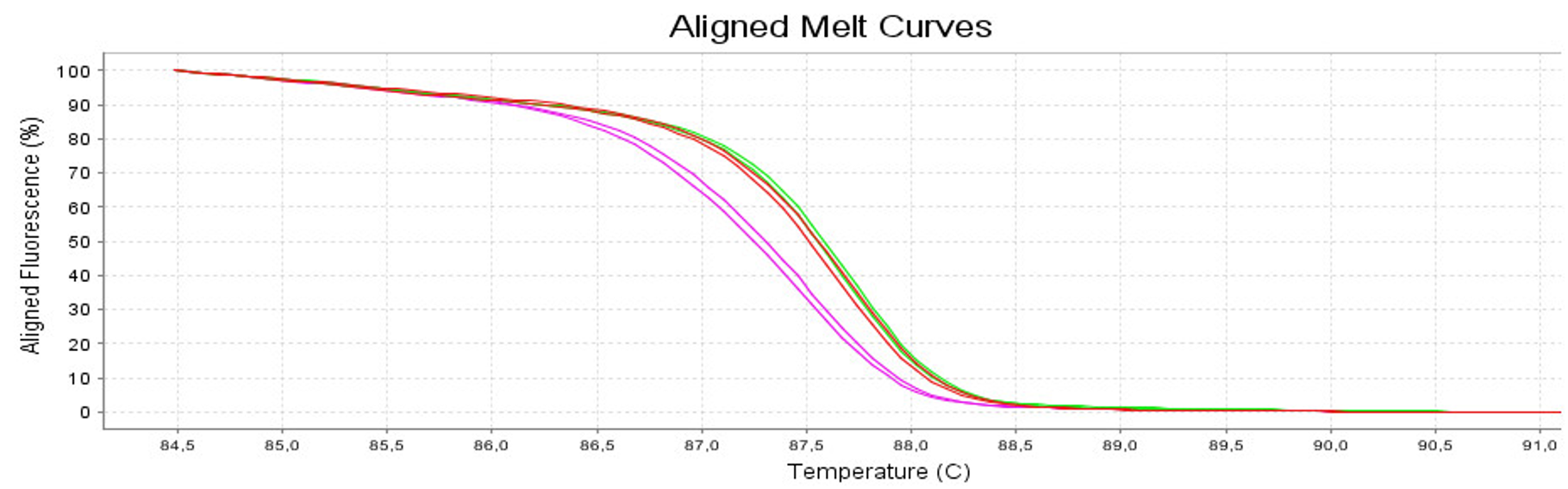

Supplement: S1 Fig — Green, red and purple (two runs each) denote, respectively: the wild type homozygous control, the c.367delC homozygous patient #683, an artificial heterozygote (1:1 mixed amplicons from the homozygous patient #810, and the control DNA). The melting patterns of two homozygotes were almost identical; therefore only heterozygotes were detectable in the HRM analysis. (TIFF) [file pone.0148067.s001.tiff]
