# Supplementary material for: ZMYND10 - Mutation Analysis in Slavic Patients with Primary Ciliary Dyskinesia
Source: PLoS One. 2016 Jan 29;11(1):e0148067. doi: 10.1371/journal.pone.0148067 (PMC4732763; doi:10.1371/journal.pone.0148067)
Supplement: S1 Table — Upper- and lowercase indicate sequences in exons and introns, respectively; asterisks denote primers pairs used in multiplex PCR with the cDNA templates (DOC) [file pone.0148067.s002.doc]

**S1 Table. List of primers. Upper- and lowercase indicate sequences in exons and introns, respectively; asterisks denote primers pairs used in multiplex PCR with the cDNA templates.**

| **Gene name** | **Primer name** | **Primer sequence** | **Product length** | **Assay** |
| --- | --- | --- | --- | --- |
| ***ZMYND10*** | ZM10_e1m1F | GAGACCTGGAACTGCTGCT | 97 bp | HRM, sequencing |
|  | ZM10_e1m1R | gtgcctcacCCTTCGGAG |  |  |
|  | ZM10_e2m2F | ccatccccactcctaatgca | 103 bp | HRM, sequencing |
|  | ZM10_e2m2R | GGCTTGCATGTTCAGCTTCT |  |  |
|  | ZM10_e1m1F | GAGACCTGGAACTGCTGCT | 133 bp | HRM, sequencing |
|  | ZM10_e2m2R | GGCTTGCATGTTCAGCTTCT |  |  |
|  | ZM10_e9m3F | AGAGTTTCCTGGCCCATCTG | 122 bp | HRM, sequencing |
|  | ZM10_e9m3R | aggtgctggtaaagtgggac |  |  |
|  | ZM10_e10m4F | GCTGGAGCGAGAAAACAGAG | 122 bp | HRM, sequencing |
|  | ZM10_e10m4R | gccatttccacaggccttac |  |  |
|  | ZM10_e3m5F | AGTGGAGATGTGGAAGCAGA | 112 bp | HRM, sequencing |
|  | ZM10_e3m5R | gtatgaaccaggggcccag |  |  |
|  | ZM10_e4m6F | ggagagctggtagcccctat | 206 bp | HRM, sequencing |
|  | ZM10_e4m6R | ttctgtgtctgatgccttcc |  |  |
|  | ZMYND_1F | aactgtcctgtcccagacttt | 277 bp | SSCP, sequencing |
|  | ZMYND_1R | gtaatactcctgtctcggaac |  |  |
|  | ZMYND_2F | caggatcaggttaggcttca | 343 bp | SSCP, sequencing |
|  | ZMYND_2R | gaaacatgtaacacattcctctttg |  |  |
|  | ZMYND_3F | ccctgtcctttaccctaccc | 168 bp | SSCP, sequencing |
|  | ZMYND_3R | gtatgaaccaggggcccag |  |  |
|  | ZMYND_4F | ggagagctggtagcccctat | 206 bp | SSCP, sequencing |
|  | ZMYND_4R | ttctgtgtctgatgccttcc |  |  |
|  | ZMYND_5F | gcaggaagggagattgtgtc | 244 bp | SSCP, sequencing |
|  | ZMYND_5R | cccagacctaggctttcaca |  |  |
|  | ZMYND_6F | aaagcctaggtctgggttcc | 208 bp | SSCP, sequencing |
|  | ZMYND_6R | cttccagccctccacagtag |  |  |
|  | ZMYND_7F | tactgtggagggctggaaga | 305 bp | SSCP, sequencing |
|  | ZMYND_7R | gaggcatcctggtatcctca |  |  |
|  | ZMYND_8F | attcaacgtctgcacccagt | 293 bp | SSCP, sequencing |
|  | ZMYND_8R | ataaacagcagggacgcagt |  |  |
|  | ZMYND_9F | cagcacctgcatcacactg | 228 bp | SSCP, sequencing |
|  | ZMYND_9R | gcaggaaggtgctggtaaag |  |  |
|  | ZMYND_10F | tgccactctccacttctctct | 255 bp | SSCP, sequencing |
|  | ZMYND_10R | ggggaaagtcaggaaggtct |  |  |
|  | ZMYND_11F | cagggccagtcagaccttc | 195 bp | SSCP, sequencing |
|  | ZMYND_11R | gggcttagaggtccaaggtt |  |  |
|  | ZMYN_c2F(*) | AGAAGCTGAACATGCAAGCC | 152 bp | RT-PCR |
|  | ZMYN_c3R(*) | GCAGAACACAGGGAACACCT |  |  |
| ***GAPDH*** | GAPDH_c4F(*) | ACGGGAAGCTTGTCATCAAT | 382 bp | RT-PCR |
|  | GAPDH_c8R(*) | GGGCCATCCACAGTCTTCT |  |  |
| ***CFTR*** | CFTR_c10F(*) | gGGATTTGGGGAATTATTTGA | 301 bp | RT-PCR |
|  | CFTR_c11R(*) | CTTTAATGGTGCCAGGCATA |  |  |
| ***LRRC6*** | LRC6_c5BF(*) | AACGAGCCAAACTCAAGGAA | 224 bp | RT-PCR |
|  | LRC6_c6R(*) | GGGCTTATTCCAGAATTCCA |  |  |
