# Supplementary material for: ZMYND10 - Mutation Analysis in Slavic Patients with Primary Ciliary Dyskinesia
Source: PLoS One. 2016 Jan 29;11(1):e0148067. doi: 10.1371/journal.pone.0148067 (PMC4732763; doi:10.1371/journal.pone.0148067)
Supplement: S2 Table — ppb, parts per billion. (DOCX) [file pone.0148067.s003.docx]

**S2 Table. Clinical data for *ZMYND10* mutated patients.** ppb, parts per billion

| **Patient** | **Situs** | **Nasal NO** | **TEM** |
| --- | --- | --- | --- |
| 683 | *Situs inversus totalis* | 117 ppb | 42 cross-sections – all lack of ODA/IDA |
| 810 | *Situs solitus* | 23; 29 ppb | 43 cross-sections – all lack of ODA/IDA |
